# Waveguide-Mode Sensors as Aptasensors

**DOI:** 10.3390/s120202136

**Published:** 2012-02-15

**Authors:** Subash C. B. Gopinath, Koichi Awazu, Makoto Fujimaki

**Affiliations:** Electronics and Photonics Research Institute, National Institute of Advanced Industrial Science and Technology, 1-1-1 Higashi, Tsukuba, Ibaraki 305-8562, Japan; E-Mail: k.awazu@aist.go.jp

**Keywords:** waveguide, aptamer, aptasensor, optics, SELEX

## Abstract

Aptamers are artificial nucleic acid ligands that can be generated by *in vitro* selection through partition and amplification. Aptamers can be generated against a wide range of biomolecules through the formation of versatile stem-loop structures. Because aptamers are potential substitutes for antibodies and drugs, the development of an aptamer-based sensor (aptasensor) is mandatory for diagnosis. We previously reported that waveguide-mode sensors are useful in the analysis of a wide range of biomolecular interactions, including aptamers. The advantages of the waveguide-mode sensor that we developed include physical and chemical stability and that higher sensitivity can be achieved with ease by perforating the waveguide layer or using colored materials such as dyes or metal nanoparticles as labels. Herein, we provide an overview of the strategies and applications for aptamer-based analyses using waveguide-mode sensors.

## 1. Introduction

A biosensor, which is a combination of a biological component with a physicochemical detector, is an analytical device that assists in the detection of biomolecular interactions. A biosensor permits investigators to detect extremely small amounts of biochemical agents, such as proteins, nucleic acid or bio-mimetic polymers, in a biological medium. Biosensors may also be utilized to analyze affinity of antibody-antigen reactions and ligand bindings. Biomolecular interactions are essential in several fields, including the clinical, diagnostic, bio-nanotechnology, molecular recognition, and medical fields. Therefore, a multitude of methods for the detection of biomolecular interactions have been developed [1–13]. The core design for sensors is mainly composed of three components, namely probe-target recognition, signal transduction, and physical readout [14]. Attempts have been made for the development of the recognition elements and aptamers have become one of the most effective recognition molecules [15]. Signaling can be achevied by labeling analytes with fluorophores, luminophores, enzymes or nanomaterials [16]. However, non-labeling methods are sometimes preferable for the realtime observation. In addition to the sensitivity, the portability and high reliability of biosensors make them very attractive for observation and detection. Some of the most widely used sensors are surface plasmon resonance (SPR)–based sensors. SPR sensor systems, which are well-known as a non-labeling detection scheme, have been proposed for the analysis of biomolecular interactions; these systems employ different surface activation, modification, and immobilization procedures on a chip. Numerous alternative sensors for biomolecular interaction analyses comparable to the SPR sensor have also been developed, one of which is the waveguide-mode sensor. Waveguide-mode sensors have been shown to have appealing characteristics such as high sensitivity, small sample requirement, real-time measurement capability [10,17–19]. The surfaces of the waveguide-mode sensors that we developed are made up of silica [10,20–22], which has suitable characteristics for biomolecular interaction analyses [11]. In the present review, we describe the application of a waveguide-mode sensor to the analysis of biomolecular interactions with aptamers.

## 2. Waveguide-Mode Sensors

A waveguide-mode sensor works in a manner similar to a SPR sensor, except that the measurement is performed using a waveguide mode rather than a surface mode [19]. The Kretschmann configuration is the most popular optical setup for both types of sensors [23]. In general, in the SPR sensor, a metal film is deposited on the surface of a glass substrate, and a prism made from the same glass is placed on the opposite side of the surface with the metal film. Usually Au or Ag is used for the metal film because SPR can be excited in these materials using visible light. In the Kretschmann configuration, when the metal layer is illuminated with light through the prism under the total reflection condition, the surface plasmons on the metal film surface are excited by the light illuminated at a specific incident angle, and the incident light is absorbed by these surface plasmons. This causes a significant decrease in the intensity of the reflected light. Because surface plasmons are sensitive to a change in permittivity at the metal surface, the resonance angle changes when biomolecules are adsorbed on the surface, and the adsorbed biomolecules can be determined from the change in the intensity of the reflected light [10,17–20,22].

**Figure 1.**
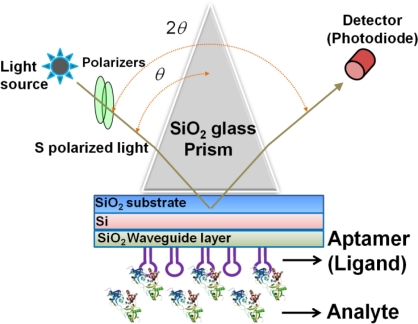
Setup for using waveguide-mode sensor as aptasensor. A triangle SiO_2_ glass prism is placed on the surface of the SiO_2_ substrate. The light illuminates the sensing plate through the prism and the reflected light is detected by the detector. The aptamer is immobilized on the surface of the sensing plate. The analytes are in the solution.

The sensing plate used in the waveguide-mode sensor has a reflective film and a transparent dielectric waveguide on the substrate glass. When light enters via the prism, coupling occurs with a waveguide mode that propagates the waveguide layer and the incident light at a certain angle of incidence. If light enters at an angle that is close to this specific angle, the intensity of the reflected light decreases significantly. Similar to the SPR mode, the waveguide mode is sensitive to the surface condition. Therefore, when biomolecules are adsorbed onto the waveguide surface, the angle at which the coupling occurs changes and correspondingly the intensity of the reflected light changes. The waveguide-mode sensor uses these changes in the intensity of reflected light to detect molecule adsorption. Previously, Au or Ag was used as the material for the reflective film. However, these materials easily peeled off from the substrate thus causing the sensing plate to become fragile. Recently, our group established a waveguide-mode sensor using Si for the reflective layer [17,19,21]; this sensor was more stable than those using Au or Ag as the reflective layer. The abovementioned waveguide-mode sensor system irradiates either s- or p-polarized light on the sensor plate. However, s-polarization was reported to show higher sensitivity than p-polarization [10]. Figure 1 shows the optical configuration of a waveguide-mode aptasensor that uses the Kretschmann configuration, wherein a sensing plate composed of a SiO_2_ substrate, Si reflective layer, and SiO_2_ waveguide is used.

It has been reported that the sensitivity of a waveguide-mode sensor is enhanced by forming nano-perforations in the waveguide layer [20]. In addition, labeling analytes with colored materials is also an effective method for enhancing the signals. The detection of molecules of various sizes has been demonstrated using biomolecular interactions and assembly processes [9,10,17,19,22]. Our group explored the use of a waveguide-mode sensor as an aptasensor with several surface modifications, surfaces with nano-perforations, and a color-labeling technique.

## 3. Aptamers

**Figure 2.**
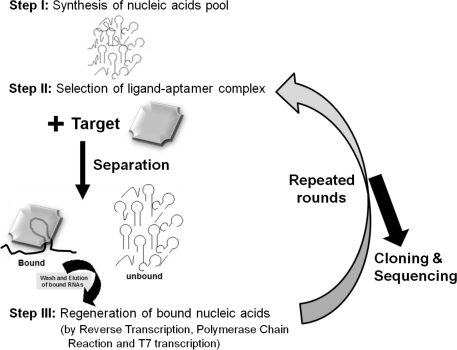
Representation of SELEX process. A specific molecule is selected from a randomized library using separation and regeneration processes. Nucleic acid pool sizes are generally ranging from 25 to100 bases. The selection procedure separates low-affinity binders and the regeneration procedure increases the number of the bound nucleic acids. The separation method is critical to remove non-specific binders.

Aptamers are unique molecules that have been proposed as substitutes for antibodies. Aptamers can behave like antibodies and have advantages over antibodies, including better stability, no batch variation, smaller sizes, and easier modification. Aptamers can be generated under laboratory conditions using randomized molecule libraries. This system involves the selection of the best molecules from the libraries against a target of interest using separation and regeneration processes [24] (Figure 2). In 1990, three different teams initially established methods for the generation of aptamers against T4 DNA polymerase and organic dyes, and selection of an RNA enzyme [25–27]. Since the invention of the systematic evolution of ligands by exponential enrichment (SELEX), this *in vitro* selection method has expanded to the fields, including molecular biology, molecular evolution, and molecular recognition to study the functional and structural aspects of nucleic acid ligands. Aptamers have been selected against a wide range of important targets for application in several fields of interest, and they have been playing a major role on the clinical front for therapy to prevent and treat disorders. The first therapeutic aptamer was commercialized in 2004. This was an aptamer generated against the vascular endothelial growth factor for the treatment of all types of neovascular age-related macular degeneration [28]. All applications that use aptamers are highly connected with sensors for diagnostic and imaging purposes. The sensors that have been developed with aptamers as biorecognition elements are called “aptasensors.”

## 4. Features of Aptamers for Aptasensors

**Figure 3.**
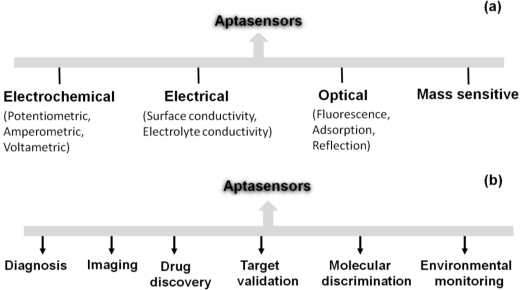
(**a**) Classification for aptasensors; (**b**) Aptasensor applications with a wide range of fields.

After the invention of aptamers in the 90s, many types of aptamer-based sensors were devised and and these devices were used in several interdisciplinary scientific applications. Aptamers are structurally versatile because they have basic stem-loop arrangements that form proper three-dimensional structures. These structures facilitate the formation of a complex with the target molecule to influence the target’s function. Aptamers have high affinities to their targets, with dissociation constants at the low-picomolar level, comparable to or better than antibodies [29]. Aptamer-based high discrimination was achieved using an anti-theophylline aptamer that discriminated caffeine from theophylline by over 10,000 fold, even though the caffeine molecule differs from theophylline only by the presence of a methyl group at the N7 position [30]. An anti-l-arginine RNA aptamer was also shown to have the ability to discriminate l-arginine from d-arginine with 12,000-fold discrimination ability [31]. Another example of selective discrimination was exhibited by an RNA aptamer selected against the cofactor nicotinamide, whereby the selected aptamer could discriminate with high accuracy between the oxidized and reduced forms of nicotinamide [32]. Similarly, in the past, aptamers have been used as single probes to efficiently discriminate between closely related proteins [33], peptide enantiomers [34], and the phosphorylated and non-phosphorylated forms of a protein [35]. Aptamers can also distinguish between closely related viral sub-types [36,37] and clotting factors [38]. These discrimination abilities have led to the development of high-performance sensors, using aptamers as the biorecognition elements. These aptasensors include electrochemical, electrical, chemiluminescence, fluorescence, quantum dot-based, colorimetric, mass spectroscopic detections [39] and are classified according to the detection mechanisms in Figure 3(a).

Aptasensors can be generated by immobilizing aptamer or partner molecules on a sensor surface [40]. Some of the designed strategies are associated with fluorescence-tagged aptasensors, including signaling by a single fluorophore, fluoreophore-quencher pair, structure-switching, and fluorogenic reaction [40]. The fluorescence labeling of an aptamer at either the 5′ or 3′ end can be done using fluorescent molecules such as fluoresceins (FAM, FITC), rhodamines (TRITC, TAMRA), cyanines (Cy3, Cy5). Baldrich *et al*. summarized the fluorescence-based assays generated for thrombin binding aptamers and these assays were demonstrated with the detection limit of sub-picomolar levels [1]. Liu and Lu have formulated an aptamer-based colorimetric assay with visible colour changes [41]. A SPR-based aptasensor is one of the predominantly established sensors to analyze the aptamer-ligand interactions as a lable-free sensing system and it can perform with the molecules having dissociation constants of lower picomolar [42]. Mass spectrometry analyses were performed for the development of aptamer microarray platforms to identify the biomarkers in serum samples [43]. An aptasensor using nanomaterials such as gold, silica, magnetic nanoparticles, carbon naontubes and polymers were reviewed by Yang *et al*. [44]. Recently, based on spectroscopic and electrochemical detection, attempts were made to compare 5′ and 3′ thiolated aptamers on porous gold substrates and it showed higher sensivity compared to the thin gold film substrates [45]. Aptamers are also harnessed in the assay known as Aptamer Linked Immunosorbent Assay (ALISA), analogous to the same format Enzyme Linked Immunosorbent Assay (ELISA) [46,47]. Enzyme-linked oligonucleotide assay (ELONA) and the mixed ELISA/ELONA assays were generated with the detection limit of ∼1 nM using thrombin aptamer [1]. The developed aptasensors are widely in use towards the future sensor developments in many fields (Figure 3(b)). Similarly, waveguide-mode sensors have been used as aptasensors to analyze aptamer-ligand interactions in a labeled or label-free manner. In the next section, we describe the use of waveguide-mode sensors as aptasensors.

## 5. Waveguide-Mode Aptasensors

**Figure 4.**
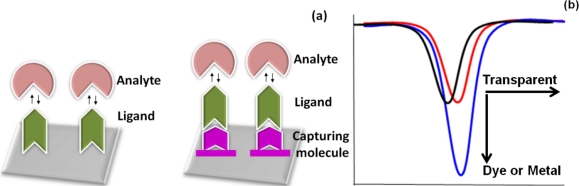
(**a**) Strategies for analyte and ligand interactions. Both direct and indirect immobilizations of the ligand on the chip are shown. The indirect immobilization requires appropriate chemical compounds as a linker; (**b**) Typical spectral changes for a waveguide-mode sensor. Horizontal (for transparent molecules) and vertical (for dyed or colored materials) changes are shown.

Aptasensors are used for analyzing biomolecules. The aptamer can be a ligand when a mixture contains an analyte, whereas the aptamer is an analyte if it is in the sample that is being analyzed. Usually, ligands are the interacting molecules immobilized on the sensor surface. Similar to other sensor surfaces, on a waveguide-mode sensor surface, the ligand can be immobilized directly on the sensor surface or indirectly via an immobilized surface chemical linkage (Figure 4(a)). The direct immobilization of molecules is mainly the result of electrostatic interaction, hydrogen bonding or van der Waals force. The direct adsorption of the molecules on the sensor surface leads to rapid, simple, and cheaper strategies compared to immobilization by chemical means. For the immobilization of aptamers through chemical linkages, any general strategy can be used because aptamers are easily modified with linkers. The selection of the linker depends on the substrate being used in the sensor. Examples of chemical-linker-based immobilization strategies for the waveguide-mode sensor surface are discussed later in this review (Section 6).

As mentioned above, in an aptasensor, the aptamer can be either a ligand or analyte, and aptamer-ligand interactions should be carried out under appropriate conditions to prepare the complex. To detect an aptamer in a waveguide-mode sensor, we carried out analyses in two different pathways based on the affinities of the aptamers against the analyte. For molecules with higher affinity, the aptasensor was used without any labeling, whereas for low-affinity molecules or a low abundance of target molecules, labeling with dye or metal nanoparticles was performed for signal enhancement. The signals of these interactions could be enhanced by using larger-sized gold nanoparticles [17]. In the case of the adsorption of non-labeled transparent molecules, adsorption could be detected as a horizontal shift in the reflection spectrum, whereas dye or metal-nanoparticle labeling resulted in spectral changes along the vertical direction (Figure 4(b)) [17,20].

## 6. Strategies for Immobilization of Aptamers on Waveguide-Mode Sensing Plates

**Figure 5.**
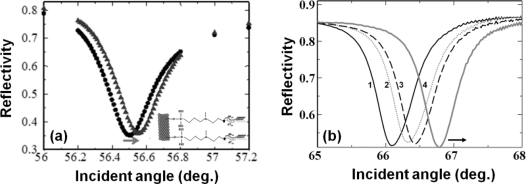
Spectral changes in waveguide-mode sensor with aptamer-based recognition. (**a**) Aptamer and factor IXa interactions on sensor surface modified with N-(2-trifluoroethanesulfonatoethyl)-N-(methyl)-triethoxysilylpropyl-3-amine (NTMTA) [20]. The inset explains the mode of aptamer attachment; (**b**) Aptamer and factor IXa interactions on nano-perforated sensing plate. The numbers 1, 2, 3, and 4 represent the different concentrations of factor IXa (0, 100, 500, and 1,000 nM, respectively) [18].

In the past, chemical immobilization strategies for aptamers have been implemented on surfaces with gold, silicon, glass, and polymer [48]. In an aptasensor, when the aptamer acts as a ligand, it requires either 5′ or 3′ end-modification to immobilize it on a solid support [49]. These modifications rely on the type of chemistry, type of spacer, spacer length, orientation of the molecules, availability of desired ends in the aptamers, and so on [50,51]. In previous studies, various surface modifications have been proposed for attaching biomolecules on silica surfaces to allow for biomolecular interactions. These include the attachment of a modified biotin through amino-coupling [17,52,53], biotin-streptavidin-biotin sandwiching on an amino surface [20,22], use of N-(2-trifluoroethanesulfonatoethyl)-N-(methyl)-triethoxysilylpropyl-3-amine-linked oligonucleotides for duplex formation [11,14], attachment of proteins to amino couplings through glutaraldehyde [21,19,54], antibody-protein-antibody sandwiching on amino-coupled glutaraldehyde [21], and thiol coupling through amino and sulfo-EMCS linkers [13,18]. Similarly, the immobilizations of biotinylated or amine-labeled aptamers on silica-based surfaces have been carried out for cancer cell detection [55]. In the past, we explored these chemical modifications on silica sensor surfaces to attach aptamers for biomolecular interactive analyses with the aid of waveguide-mode sensors. In one strategy, the aldehyde at the ends of glutaraldehyde, followed by streptavidin, was attached to the amino surfaces in order to capture biotin molecules with 20 bases of deoxythymines; thereafter, further duplexes were prepared using an anti-factor IXa aptamer with an extended tail. The biotin molecules were attached at the 5′ end, and the complementation was made at the 3′ end. These bio-molecular assembly processes mediated by the aptamers were used to monitor the interactions for factor IXa and factor IX binding proteins [10]. We also performed experiments to capture and analyze cyanocobalamin using an anti-cyanocobalamin aptamer [20]. In another type of surface chemical modification, to initially attach the aptamer, the compound NTMTA was prepared with deoxythimines, after which the factor IXa aptamer was duplexed using the extended 3′ end of the aptamer with polyadenines and used to investigate the target protein binding (Figure 5(a)).

Perforated sensor surfaces are attractive because the perforations increase molecular accommodation [56]. Various sensor surfaces made of alumina, polymer, and silicon with nano-perforations for waveguide-based sensors have been reported [12,57–59]. These nano-perforations on sensor chips are useful for the development of label-free aptasensors with high sensitivity. The sensitivity of sensors having surfaces with nano-perforations relies on the density, depth, and size of the nano-perforations. However, a nano-perforation diameter larger than the diffraction limits causes scattering of incident light and interference, resulting in broadening of the resonance signals. Our team created nano-perforations on waveguide-mode sensor plates with a density of 1.0 × 10^10^ cm^−2^ and perforation size of 50 nm [53]. We determined the effect of a perforated silica film against a bulk film and found a nine-fold increase in the sensitivity with the perforated sensor plate. In addition, it has been reported that nano-perforations with diameters of around 50 nm are suitable for a waveguide-mode sensor operated under visible light [20]. Rong *et al.* quantified the attachment of molecules inside 20-nm-diameter nano-perforations on a silicon-based waveguide-based sensor by monitoring the resonance changes caused by the complementation of DNA molecules [12]. On a sensor chip surface with nano-perforations, we modified the surface using sodium (1-{[6-(2,5-dioxo-2,5-dihydro-1*H*-pyrrol-1-yl)hexanoyl]oxy}-2,5-dioxopyrrolidine-3-sulfonate (sulfo-EMCS) as the cross-linking agent to facilitate the attachment of aptamers [18]. To attach the aptamer to the sulfo-EMCS modified surfaces through an amino linker, we initially attached the thiolated deoxythimines with 20 bases to the sulfo-EMCS groups. Then, the anti-factor IXa aptamer having an extended tail with adenine bases was complexed. The resulting surface could be cleaned for reuse simply by changing the pH of the buffering solution. This surface permitted the analysis of the aptamer against the factor IXa protein. In addition, because the sensor surface was reusable, it was used to determine the concentration-dependent binding of factor IXa against the aptamer (Figure 5(b)). These results suggest that in addition to surface chemical modifications, perforating a sensing surface is good for increasing its sensitivity without modifying the properties of the material used for preparing the sensor surface.

## 7. Signal Enhancement for Aptamer-Protein Interactions

**Figure 6.**
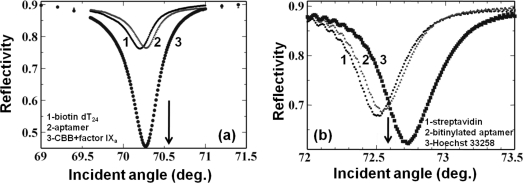
Effect of dye on spectral changes in waveguide-mode sensor. (**a**) CBB-based signal enhancement with aptamer and factor IXa [22]; (**b**) Interactions of DNA-aptamer and Hoechst dye [22].

To improve the sensitivity of a waveguide-based aptasensor, we used a dye, Coomassie Brilliant Blue (CBB), conjugated on the target proteins. In the analyses, we examined two aptamers generated against rabbit IgG [60] and factor IXa [61]. In both cases, biotin-steptavidin-biotin sandwiching was performed to attach the biotinylated aptamers based on the amino surface of the sensing plate as described above, through the attachment of aptamer sequences on the biotin extended with 20-base oligos (dT20). This Watson-Crick duplex formation between the aptamer and oligo (dT20) acted as a ligand. Then, we passed a CBB-target protein complex dissolved in buffers with appropriate components over this aptamer-immobilized surface to obtain increased signal. In this case, the obtained sensitivity was 50 times higher than that of a CBB-less strategy [21] (Figure 6(a)). We also analyzed the previously-generated DNA aptamer against the dye Hoechst 33258 [62], and the binding events where the dye could enhance the signal were monitored. In this case, because DNA can be easily modified with biotin, we synthesized an anti-Hoechst 33258 aptamer using biotin at the 5′ end and formed a biotin-streptavidin-biotin complex to analyze the aptamer-target interactions as described above. In this case, we obtained a signal enhancement, even though the enhancement factor was smaller than that obtained with CBB (Figure 6(a,b)). Mutation of the Hoechst aptamer with GC bases by replacement of the essential AT regions caused significant reduction in the reflectivity changes [22]. All of the above aptamer and target interactions analyses are summarized in Table 1. Based on the ligand (aptamer)-analyte (target) affinities, different aptamers show different changes in angular shift or reflectivity. In all of the above interactions listed in Table 1, aptamer concentrations of 100 to 800 nM were maintained to maximize the possibility of their immobilization on the sensor surface. On these aptamer-attached surfaces, the appropriate target molecules (1 to 1,000 nM) were analyzed to differentiate the effects of chemically modified sensor surfaces. The reaction buffers used for all interactions include NaCl and MgCl_2_ or NaCl and CaCl_2_, as originally reported [60,61]. Further, signal enhancement could also be achieved using gold nanoparticle–conjugated biomolecules [17].

## 8. Compact Waveguide-Mode Aptasensor

**Figure 7.**
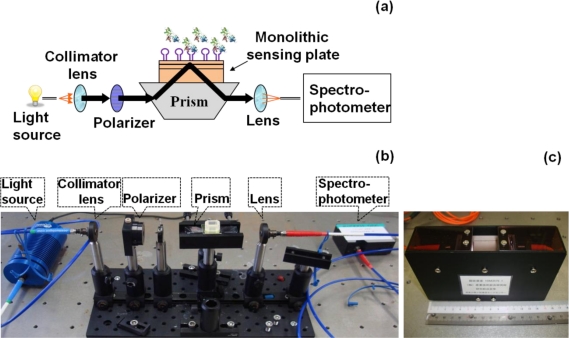
Representations of compact waveguide-mode sensor system: (**a**) diagrammatic representation; (**b**) photograph of the setup; and (**c**) photograph of the handy portable prototype of the sensing system. The spectral readout system is employed in the setup.

**Figure 8.**
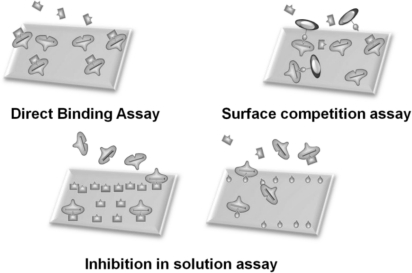
Proposed assays on waveguide-mode sensor using aptamer and ligands. These assays can be designed according to the experimental plan.

In general, sensor systems are expected to be highly sensitive, precise, portable, easy to use, and inexpensive. The waveguide-mode aptasensor shown here can satisfy all abovementioned requirements. As previously reported, in the label-free scheme, the sensitivity of the waveguide-mode sensor is 4 times better than that of the SPR sensor. In addition, if the perforated sensing plate is used, the sensitivity becomes 5 to 10 times better than that of the waveguide-mode sensor that uses the non-perforated sensing plate [63]. Furthermore, if color-labeling technique is applied, the waveguide-mode sensor is able to detect 10-pM biotin-streptavidin interactions [17]. Accordingly, we can say that the waveguide-mode sensor has good sensitivity. To develop a waveguide-mode aptasensor that is handy portable and easy to use, we recently developed a compact portable system that employs a spectral readout system. The optical arrangement of this waveguide-mode sensor is shown in Figure 7(a,b). Figure 7(c) shows a prototype of the sensor that is handy portable. A similar system was proposed by Bolduc *et al.*, for use with wavelength-interrogation surface plasmon resonance spectroscopy using a dove prism for high-resolution analyses [64]. Similar to other systems, by using aptamer and target interactions on a waveguide sensor chip, we can employ various types of assays, including direct binding assays, surface competition assays, and aptamer-based inhibition assays (Figure 8). These assays with an aptamer–ligand complex and drug or chemical libraries will promote the waveguide-mode sensor system as the ideal sensor for drug discovery. An aptamer-ligand complex can be attached to the sensing plate. Then, upon the passage of a candidate drug as an analyte over this complex, the aptamer would be replaced if the drug has more potential than the aptamer.

## 9. Future Prospects

Specific aptamer recognitions have been proposed in several sensor developments, with their participation in multiple disciplines. Applications of aptamers in the medical and immunodiagnostic fields have suggested that an aptasensor could be an alternate to other sensors in nanotheranostics. Aptamers can be used to analyze nucleic acid-ligand interactions, to generate inhibitors to be used for pharmacological purposes, to detect a target in a complex mixture, and to generate the lead components in medicinal chemistry. However, the development of an effective aptamer is a time-consuming task and requires large effort. Therefore, aptasensor is expected to be a faster and easier screening method for aptamer generation. The applications of aptamers (so-called “chemical antibodies”) in combination with waveguide-mode sensors are proposed to analyze small molecules and proteins with a view to applying this sensor to a wide range of biomolecular interactive analyses. The waveguide-mode aptasensor, which can detect molecules without labeling, will provide an easy and fast screening system for the aptamer development. However, the detection limit of the non-labeling way is in the order of nanomolar, which is sometimes not enough. Further increment of the sensitivity will be the future subject. Taking all of the above-described appealing points into account, we opine that an aptamer-based waveguide-mode sensor is one of the best substitutes for the currently available antibody-based sensors.

## Figures and Tables

**Table 1.** Aptamer recognitions on waveguide-mode sensors.

**Surface chemistry**	**Ligand (aptamer)**	**Analyte**	**Conc. of Aptamer (nM)**	**Conc. of Analyte (nM)**	**Mode**	**Angular shift**	**Reflectivity**	**References**

Sulfo-EMCS	RNA	Factor IXa	800	100	without dye	0.23°	-	[18]
Streptavidin-Biotin	RNA	Factor IXa	100	100	without dye	0.12°	-	[10]
Biotin-streptavidin-biotin	RNA	Cyanocobalamin	500	1000	without dye	0.27°	-	[20]
NTMTA	RNA	Factor IXa	500	500	without dye	0.06°	-	[20]
Biotin-streptavidin-biotin	RNA	Rabbit IgG	100	1	with dye	-	0.3 *	[22]
Biotin-streptavidin-biotin	RNA	Factor IXa	100	1	with dye	-	0.33 *	[22]
Biotin-streptavidin-biotin	DNA	Hoechst 33258	500	10	with dye	-	0.07 **	[22]

* CBB was used for signal enhancement;

** Hoechst was used as the dye. In both cases, changes in reflection along the vertical direction were measured.
